# Supplementary material for: The relationship between wellness and training and match load in professional male soccer players
Source: PLoS One. 2023 Jul 31;18(7):e0289374. doi: 10.1371/journal.pone.0289374 (PMC10389715; doi:10.1371/journal.pone.0289374)
Supplement: S1 Table — MD: Match day; MD+1: One day after the match day); MD-5: Five days before match day; MD-4: Four days before match day; MD-3: Three days before match day; MD-2: Two days before match day; MD-1: One day before match day; RPE: Rate of perceived exertion using the CR-10 Borg’s scale; Session-RPE: Multiplication of time of session by the score of RPE; A.U.: Arbitrary units; m: Meters; min: Minutes; nr, number; HSR: High speed running distance (20–25 km/h). (DOCX) [file pone.0289374.s001.docx]

**S1 Table . Effect sizes of the comparisons presented in table 2.**

| Variables | Effect size |
| --- | --- |
| Quality of sleep (A.U.) | MD+1 vs MD-5: -2.19  MD+1 vs MD-4: -2.45  MD+1 vs MD-3: -2.66  MD+1 vs MD-2: -3.80  MD+1 vs MD-1: -3.00 |
| Fatigue (A.U.) | MD+1 vs MD-5: -1.43  MD+1 vs MD-4: -1.12  MD+1 vs MD-3: -1.12  MD+1 vs MD-2: -1.12  MD+1 vs MD-1: -1.61  MD+1 vs MD: -1.74  MD+1 vs MD: -0.76  MD-4 vs MD: -0.76  MD-3 vs MD: -0.76  MD-2 vs MD: -0.76 |
| Muscle Soreness (A.U.) | MD+1 vs MD-5: -0.86  MD+1 vs MD-4: -0.81  MD+1 vs MD-1: -1.12  MD+1 vs MD: -1.27  MD-4 vs MD: -1.27  MD-3 vs MD: 0.32  MD-2 vs MD: 0.57  MD-1 vs MD: 0.19 |
| Stress (A.U.) | MD+1 vs MD-5: -0.59  MD+1 vs MD-4: -0.39  MD+1 vs MD-3: -0.39  MD+1 vs MD-2: -0.39  MD+1 vs MD-1: -0.50  MD+1 vs MD: -0.50 |
| Mood (A.U.) | MD+1 vs MD-5: -1.26  MD+1 vs MD-4: -0.81  MD+1 vs MD-3: -0.81  MD+1 vs MD-2: -0.81  MD+1 vs MD-1: -0.81  MD+1 vs MD: -0.81 |
| RPE (A.U.) | MD+1 vs MD-4: -2.56  MD+1 vs MD-3: -2.16  MD+1 vs MD: -2.13  MD-5 vs MD-4: -2.35  MD-5 vs MD-3: -1.26  MD-5 vs MD-2: 2.85  MD-5 vs MD-1: 4.10  MD-5 vs MD: -1.40  MD-4 vs MD-2: 4.75  MD-4 vs MD-1: 5.54  MD-3 vs MD-2: 3.82  MD-3 vs MD-1: 4.97  MD-2 vs MD-1: 2.08  MD-2 vs MD: -2.45  MD-1 vs MD: -3.51 |
| Session duration (min) | MD+1 vs MD-4: -1.63  MD+1 vs MD: -3.11  MD-5 vs MD-4: -2.84  MD-5 vs MD-2: 1.87  MD-5 vs MD-1: 7.15  MD-5 vs MD: -3.57  MD-4 vs MD-3: 1.58  MD-4 vs MD-2: 2.92  MD-4 vs MD-1: 5.81  MD-4 vs MD: -2.70  MD-3 vs MD-2: 2.32  MD-3 vs MD-1: 7.20  MD-3 vs MD: -3.42  MD-2 vs MD-1: 4.64  MD-2 vs MD: -3.91  MD-1 vs MD: -4.94 |
| s-RPE (A.U.) | MD+1 vs MD-4: -1.55  MD+1 vs MD-3: -1.16  MD+1 vs MD: -4.06  MD-5 vs MD-4: -4.70  MD-5 vs MD-3: -2.41  MD-5 vs MD-2: 4.28  MD-5 vs MD-1: 9.67  MD-5 vs MD: -9.88  MD-4 vs MD-3: 1.06  MD-4 vs MD-2: 4.01  MD-4 vs MD-1: 6.59  MD-4 vs MD: -5.60  MD-3 vs MD-2: 3.52  MD-3 vs MD-1: 6.57  MD-3 vs MD: -7.06  MD-2 vs MD-1: 4.89  MD-2 vs MD: -11.41  MD-1 vs MD: -13.62 |
| HSR (m) | MD+1 vs MD-4:-1.42  MD+1 vs MD-3: -1.34  MD+1 vs MD: -3.97  MD-5 vs MD-4: -2.47  MD-5 vs MD-3: -2.25  MD-5 vs MD-2: 1.96  MD-5 vs MD-1: 3.19  MD-5 vs MD: -7.11  MD-4 vs MD-2: 5.22  MD-4 vs MD-1: 7.03  MD-4 vs MD: -5.43  MD-3 vs MD-2: 3.25  MD-3 vs MD-1: 4.40  MD-3 vs MD: -3.96  MD-2 vs MD-1: 2.15  MD-2 vs MD: -13.76  MD-1 vs MD: -16.65 |
| Sprint Distance (m) | MD+1 vs MD-4: -1.36  MD+1 vs MD: -2.11  MD-5 vs MD-4: -3.11  MD-5 vs MD-2: 2.21  MD-5 vs MD-1: 3.98  MD-5 vs MD: -3.82  MD-4 vs MD-3: 1.75  MD-4 vs MD-2: 6.99  MD-4 vs MD-1: 11.45  MD-3 vs MD-2: 3.72  MD-3 vs MD-1: 6.30  MD-3 vs MD: -2.69  MD-2 vs MD: -6.79  MD-1 vs MD: -7.10 |
| Accelerations (nr) | MD+1 vs MD-5: -1.25  MD+1 vs MD-4: -1.35  MD+1 vs MD-3: -1.46  MD+1 vs MD: -2.80  MD-5 vs MD-2: 1.44  MD-5 vs MD-1: 3.31  MD-5 vs MD: -2.52  MD-4 vs MD-2: 2.78  MD-4 vs MD-1: 6.13  MD-4 vs MD: -3.17  MD-3 vs MD-2: 2.80  MD-3 vs MD-1: 5.80  MD-3 vs MD: -2.83  MD-2 vs MD: 3.50  MD-2 vs MD-1: -4.82  MD-1 vs MD: -6.65 |
| Decelerations (nr) | MD+1 vs MD-5: -1.33  MD+1 vs MD-4: -1.52  MD+1 vs MD-3: -1.65  MD+1 vs MD: -3.38  MD-5 vs MD-2: 1.24  MD-5 vs MD-1: 3.36  MD-5 vs MD: -3.12  MD-4 vs MD-2: 2.39  MD-4 vs MD-1: 6.04  MD-4 vs MD: -3.61  MD-3 vs MD-2: 2.69  MD-3 vs MD-1: 6.30  MD-3 vs MD: -3.38  MD-2 vs MD: 4.05  MD-2 vs MD-1: -5.15  MD-1 vs MD: -7.11 |

MD: match day; MD+1: one day after the match day); MD-5: five days before match day; MD-4: four days before match day; MD-3: three days before match day; MD-2: two days before match day; MD-1: one day before match day; RPE: rate of perceived exertion using the CR-10 Borg’s scale; Session-RPE: multiplication of time of session by the score of RPE; A.U.: arbitrary units; m: meters; min: minutes; nr, number; HSR: high speed running distance (20-25 km/h).
