# Supplementary material for: The relationship between wellness and training and match load in professional male soccer players
Source: PLoS One. 2023 Jul 31;18(7):e0289374. doi: 10.1371/journal.pone.0289374 (PMC10389715; doi:10.1371/journal.pone.0289374)
Supplement: Attachment — Submitted filename: PONE-D-23-04228.docx [file pone.0289374.s002.docx]

**Dear authors:**

Overall, the study aimed to investigate within-microcycle variations of professional soccer players, analyzing the relationships between wellness and load demands, as well as the relationships between match demands and wellness of MD+1 and MD-1. The results showed that wellness variables remained similar across all sessions, except for MD+1 where lower values were found. The study also found that the lowest internal and external intensity values were found on MD-1 while the highest were found on MD. These findings are consistent with previous studies that have also found lower values on MD-1 and higher values on MD-5, MD-4, and MD-3.

The authors suggest that the reduction in wellness on MD-1 may be associated with a tapering period aimed at decreasing fatigue and increasing recovery to achieve better performance in the following match. The authors also suggest that the results of this study may help coaches and practitioners to better plan and monitor training loads and recovery strategies for professional soccer players.

However, it should be noted that the study has some limitations, such as the small sample size and the fact that only one team was studied. Therefore, further research with larger samples and multiple teams is needed to confirm the findings of this study. Additionally, future studies could investigate the impact of different tapering strategies on player performance and wellness. In the below you can find my comments to improve some sections:

**Title**
Based on the content of the study, a possible suggestion for the title could be "Relationships between internal and external load demands and wellness in professional soccer players across microcycles".

**Introduction**

It is well written. There is no comment in this section.

**Method:**

**Participant section:**

Overall, the participant section is well written and provides relevant information about the sample size, characteristics of the players, and inclusion criteria. However, as a reviewer, I would suggest adding more information about the recruitment process and how participants were selected for the study. It would also be useful to know if any players were excluded from the study and for what reasons.

Additionally, the authors could provide more information on how they ensured compliance with the eligibility criteria, such as how they tracked participation in training sessions and wellness and training intensity reports. This information would increase the transparency of the study and help readers understand the validity of the data collected.

Finally, the authors should clarify whether the power calculations were conducted prior to or after data collection. If it was done prior to data collection, it would be useful to know how the authors arrived at the effect-size of 0.2 and the correlation coefficient of 0.7 used in the calculations.

**External intensity quantification & Internal intensity quantification section:**

The methods section regarding the external and internal intensity quantification is well described and adequately detailed. However, it could be improved by providing more information on the interpretation of the results obtained through the GPS Vector S7. For instance, the authors could explain how the measured variables, such as high-speed running and sprinting, relate to the overall physical performance of the players. Moreover, it would be helpful to mention the standard deviation or range of values for each variable to provide a better understanding of the players' physical demands during training and matches. Finally, the authors could also provide information on how the CR-10 Borg’s scale is typically used to assess RPE in soccer players, and how session-RPE is calculated based on this scale.

**Discussion**

As a general view, here are a few suggestions for improving the conclusions section:

Provide more context: While the conclusions do summarize the findings of the study, they could benefit from additional context. It would be helpful to briefly explain why the study findings are significant and what implications they have for future research or practice.

Be more specific: The conclusions mention several associations between intensity and wellness variables, but they do not provide specific details or effect sizes. Including more specific information would help readers better understand the significance of these relationships.

Discuss future directions: Given the findings of the study, it would be valuable to suggest areas for future research or potential interventions that could be developed to address the identified relationships between intensity and wellness variables.
