# Supplementary material for: The relationship between wellness and training and match load in professional male soccer players
Source: PLoS One. 2023 Jul 31;18(7):e0289374. doi: 10.1371/journal.pone.0289374 (PMC10389715; doi:10.1371/journal.pone.0289374)
Supplement: Attachment — Submitted filename: Response to Reviewers.docx [file pone.0289374.s003.docx]

**Reviewer 1**

**Dear authors:**

Overall, the study aimed to investigate within-microcycle variations of professional soccer players, analyzing the relationships between wellness and load demands, as well as the relationships between match demands and wellness of MD+1 and MD-1. The results showed that wellness variables remained similar across all sessions, except for MD+1 where lower values were found. The study also found that the lowest internal and external intensity values were found on MD-1 while the highest were found on MD. These findings are consistent with previous studies that have also found lower values on MD-1 and higher values on MD-5, MD-4, and MD-3.

The authors suggest that the reduction in wellness on MD-1 may be associated with a tapering period aimed at decreasing fatigue and increasing recovery to achieve better performance in the following match. The authors also suggest that the results of this study may help coaches and practitioners to better plan and monitor training loads and recovery strategies for professional soccer players.

However, it should be noted that the study has some limitations, such as the small sample size and the fact that only one team was studied. Therefore, further research with larger samples and multiple teams is needed to confirm the findings of this study. Additionally, future studies could investigate the impact of different tapering strategies on player performance and wellness. In the below you can find my comments to improve some sections:

Authors: Thank you very much for your comments. They were very helpful.

**Title**
Based on the content of the study, a possible suggestion for the title could be "Relationships between internal and external load demands and wellness in professional soccer players across microcycles".

Authors: Considering the English proofreading, we changed the title to a similar manner.

**Introduction**

It is well written. There is no comment in this section.

Authors: Thank you very much.

**Method:**

**Participant section:**

Overall, the participant section is well written and provides relevant information about the sample size, characteristics of the players, and inclusion criteria. However, as a reviewer, I would suggest adding more information about the recruitment process and how participants were selected for the study. It would also be useful to know if any players were excluded from the study and for what reasons.

Authors: Information was added accordingly.

Additionally, the authors could provide more information on how they ensured compliance with the eligibility criteria, such as how they tracked participation in training sessions and wellness and training intensity reports. This information would increase the transparency of the study and help readers understand the validity of the data collected.

Authors: Done, thank you.

Finally, the authors should clarify whether the power calculations were conducted prior to or after data collection. If it was done prior to data collection, it would be useful to know how the authors arrived at the effect-size of 0.2 and the correlation coefficient of 0.7 used in the calculations.

Authors: The information was already presented in the original version. Specifically, “Two Post-hoc analyses were conducted considering both types of aim (comparisons and correlations). Thus, for the comparison analysis, an F-test, with a total of 13 participants with a *p* < 0.05 and effect-size of 0.2 and seven measurements (6 training sessions and 1 MD) was performed. The actual power achieved was 95.4%. Considering the correlation analysis, a Bivariate normal model with correlation of 0.7, *p* < 0.05 and the same 13 participants was applied which revealed 81.7% of actual power.” This means that power calculations were performed after the data collection.

**External intensity quantification & Internal intensity quantification section:**

The methods section regarding the external and internal intensity quantification is well described and adequately detailed. However, it could be improved by providing more information on the interpretation of the results obtained through the GPS Vector S7. For instance, the authors could explain how the measured variables, such as high-speed running and sprinting, relate to the overall physical performance of the players. Moreover, it would be helpful to mention the standard deviation or range of values for each variable to provide a better understanding of the players' physical demands during training and matches. Finally, the authors could also provide information on how the CR-10 Borg’s scale is typically used to assess RPE in soccer players, and how session-RPE is calculated based on this scale.

Authors: Thank you for the positive comments. Regarding the description of high-speed running and sprinting as the overall locomotor demands of the players, we opted to change this description to load demands which we believe it is more appropriate. Moreover, the standard deviation of each variable was presented in table 2 which contains the descriptive statistics. Finally, we add a sentence to support the use of the CR-10 Borg’s scale in soccer studies while the calculation of session-RPE was already described in the “Internal intensity quantification” section. Thank you

**Discussion**

As a general view, here are a few suggestions for improving the conclusions section:

Provide more context: While the conclusions do summarize the findings of the study, they could benefit from additional context. It would be helpful to briefly explain why the study findings are significant and what implications they have for future research or practice.

Be more specific: The conclusions mention several associations between intensity and wellness variables, but they do not provide specific details or effect sizes. Including more specific information would help readers better understand the significance of these relationships.

Discuss future directions: Given the findings of the study, it would be valuable to suggest areas for future research or potential interventions that could be developed to address the identified relationships between intensity and wellness variables.

Authors: Thank you very much for your suggestion. They were implemented accordingly.

**Reviewer #2:** Thank you to the authors for putting together this comprehensive manuscript. It is very interesting with very relevant and practical findings; however, the quality of writing needs to be improved significantly. See some comments below:

Abstract
• Indicate which measures are IL and which are EL

Authors: Done.

• Change ap-plied to “applied”

Authors: Done.

• Rephrase to: “While MD-1 presented the lowest internal (variables) and external load (variables) values, MD presented the highest values”

Authors: Done.

• When you say “time of the session” – are you referring to the time of day or session duration?
Authors: Session duration. It was changed accordingly through all text.

Intro
• Line 2: “…is a common practice to reduce to the risk of injury and overtraining.”

Authors: Done.

• Line 3: “…practitioners employ periodization strategies by adjusting training intensities considering the previous sessions.”

Authors: Done.

• Line 14: strengthen topic sentence of this paragraph –

Authors: Please check the following paragraph in which the topic is developed.

• Line 15: begin sentence with “specifically”

Authors: Done.

• Paragraph 2 needs to be strengthened with improved writing quality

Authors: A proofreading was conducted through all manuscript.

• Line 29: add a comma after session and after wellness perception

Authors: Done.

• Line 32: briefly note what the relationship outcome was “This was evident in a recent study on youth soccer players, where sleep and intensity…” ⇓ discuss in that one sentence was the main outcome was

Authors: Done.

Methods
• Design – you only mention sleep, but you monitored more wellness variables also. Either list them all here or introduce all in the later section

Authors: Done.

• Rephase: “..before each training/match session through a specifically designed google form.”

Authors: Done.

• The questionnaire uses a Likert scale ranging from 1-5, in which athletes were asked to rate their fatigue, sleep quality, muscle soreness, stress, and mood (5=very fresh, very restful, very great, very relaxed, and very positive mood, respectively; 1=always tired, insomnia, very sore, highly stressed, and highly annoyed/irritable/down, respectively). All players were already familiarized with the questionnaire from the previous season.”

Authors: Done.

• Combine lines 88 and 92 – very repetitive, just throw in the 10hz sampling session in line 88

Authors: Done.

• Lines 96-97: delete the authors from the text and just include their reference as validation

Authors: Done.

• Line 106: delete this topic sentence

Authors: Done.

• Line 108: change during to from

Authors: Done.

Results
• Table 2: add nr in the legend

Authors: Done.

• Lines 130-132: when you say wellness variables including sleep quality and fatigue, are those the only 2 wellness variables? Or are there others? This is unclear by the phrasing of the sentence. Please rephrase to be more clear
• Line 132: didn’t you just say this in the previous sentence?
• Line 133-134: again, repeating the last 2 sentences you wrote – synthesize, as this is very repetitive

Authors: The entire paragraph was revised for better clarity. Thank you for your attention.

• Even though all numbers are displayed in Table 2, please still include actual numbers in the results section. Don’t need every single number, but some means or p values or effect sizes would strengthen this section

Authors: We opted to not follow your suggestion to avoid repetitive information and a hard text to read. We hope you can understand.

• Please rephrase “time of session” to “session duration” throughout your paper

Authors: It was replaced accordingly.

• This reads moreso like a list to me – I think by adding numbers as I suggested above will help break that up.

Authors: The idea of the text is to provide more details not presented in the table 2. Adding numbers would just make difficult the reading and it would also contribute for a repetitive text. We hope that the reviewer can understand.

• For tables 4 and 5 results, why not run a regression for highly correlated variables?

Authors: We understand the reviewer’s comments. Indeed, we would like to present such a analysis. However, our sample is to small to provide a significant and powerful result. Therefore, we just present the correlations, but we added it as a suggestion for future studies.

Discussion
• Line 226: which wellness variables were lower in MD+1?

Authors: All wellness variables. We clarified this point in the sentence.

• If citing an SR, discuss/cite the studies individually

Authors: In this case, we opted to keep the original sentence considering that it a conclusion of the systematic reviews. Besides, cite all the studies would just increase the number of references of the present study which is not the best science practice. We hope the reviewer can understand.

• Appears that discussion is out of order – you discuss aim 3 before aim 2

Authors: Thank you for pointing this issue. In fact, aim 2 was not discussed and it was not added to the discussion section.

• Significant improvements in writing is needed

Authors: A proofreading was conducted in all text by one of the authors who is English native speaker and an expert in the field. Thank you

**Reviewer #3:** Congratulations to the authors. But some minor adjustments need to be made;

Authors: Thank you very much for the positive feedback.

- Hypotheses should be written more clearly and precisely.

Authors: Done.

-Line 46. “This was an observational study design” please explain and show references.

Authors: Done.

-Line 46-47, sleep quality and duration?? You can use welness measures instead of sleep quality and duration.

Authors: We amended the sentence for better clarity. Thank you

Line- 48, give more details about the content of the 70 training sessions?

Authors: The present investigation did not influence or alter the training sessions design planned by the coach. For such reason, and for the fact that the club did not allow providing such details, the previous information was added in the text.

Line 49-50, please give more details about the reseacrh design? Which microcycles were chosen?

Authors: The information was added accordingly.

-In the Methods section, Please explain which sampling method was used to create my sample group. It is based on scientific source.

Authors: Done.

-The data collection process cannot be detailed in method section. Where, by whom, and in what time of day the data were collected. It should be detailed.

Authors: The information was added in the design section. Thank you

-Please give more details about eligibility criteria.

Authors: Done.

Line 61, a European soccer team or its country? Which league?

Authors: We only mention European soccer league due to ethical reasons. The club from which the players belonged did not authorized this information. We hope that reviewer can understand.

Line 59-60, body mass, height, and fat mass? How did you determine this? Please add fat free mass also. Please add the explanations in methods section. Were these measurements made on an empty stomach or on a full stomach? What time of day was it done? Were the participants informed about the criteria recommended by the ACSM before the measurements? Show references for these all measurements and explanations.

Authors: Done.

-In the resuts section, there are sentences that repeat each other. Please revise them.

Authors: All text was revised accordingly.

-Please develop practical applications section of the study.

Authors: Done.
