# Supplementary material for: The relationship between wellness and training and match load in professional male soccer players
Source: PLoS One. 2023 Jul 31;18(7):e0289374. doi: 10.1371/journal.pone.0289374 (PMC10389715; doi:10.1371/journal.pone.0289374)
Supplement: Attachment — Submitted filename: Response to Reviewers.docx [file pone.0289374.s004.docx]

**Cover Letter**

**PLOS ONE Editorial Staff,**

**Dear Editor,**

Please find enclosure the revision of our manuscript: “The relationship between wellness and training and match load in professional male soccer players”. The revision includes three files as requested: a rebuttal letter that responds to each point raised by the academic editor and reviewer(s); a 'Revised Manuscript with Track Changes'; and an unmarked version of your revised paper without tracked changes labeled as 'Manuscript'. The answers presented in the rebuttal letter can also be find in the following text:

**Comments of the Editor**

Dear authors, thanks for the revisions made addressing both reviewers' comments. After carefully checking changes and improvements I have noticed some minor issues that should be implemented in a minor revision. Please, I encourage to revise the manuscript according to the following minor issues:

Answer: Dear editor, thank you very much for your suggestions which improve quality to our work.

- L142-143, please cite the SPSS software following the company criterion: <https://www.ibm.com/support/pages/how-cite-ibm-spss-statistics-or-earlier-versions-spss>

Answer: The citation was updated as follows: “IBM SPSS Statistics for Windows version 23.0 (IBM Corp, Armonk, NY,USA).”

- Consistency is required when using abbreviations (e.g., effect size: ES). Most of the times, the authors used “effect size” and a few times the abbreviation HA. If the intention is to use the abbreviation, on the first occasion write in full “effect size”, indicating the abbreviation in parentheses (ES). Then, always use ES.

Answer: Done. Thank you for the attention.

- In table 1 abbreviations should appear using the presentation of tables 2 and 3, W: week.

Answer: Done.

- Provide DOI to all references: e.g. Reference 15, 22 and 23

Answer: DOI’s were added accordingly.

**Comments of Reviewer 2**

Thank you for your updates to this paper. My remaining comment is for the results section, particularly for table 2. In the written text you note effect sizes, but there is no data on effect sizes in the table. Please include these numbers in the written text.

Answer: Dear reviewer, thank you for the positive feedback. Considering that adding the exact numbers of the ES would difficult the reading, we added a new table as a Supplementary table 1 to provide all ES values. Thank you.

Best regards
